# Supplementary material for: Modeling glioblastoma heterogeneity as a dynamic network of cell states
Source: Mol Syst Biol. 2021 Sep 16;17(9):e10105. doi: 10.15252/msb.202010105 (PMC8444284; doi:10.15252/msb.202010105)
Supplement: Supplementary file 6 — Source Data for Figure 5 [file MSB-17-e10105-s004.zip › Figure5A_sourcedata/GSEA_3071/nosigdb_stateII.GseaPreranked.1622795151171/RICHARDS_NOWAKOWSKI_SCIENCE_2017_MGE_DIV_UPREG.html]

Details for gene set RICHARDS\_NOWAKOWSKI\_SCIENCE\_2017\_MGE\_DIV\_UPREG[GSEA]

|  || Dataset | state23071 |
| Phenotype | NoPhenotypeAvailable |
| Upregulated in class | na\_pos |
| GeneSet | RICHARDS\_NOWAKOWSKI\_SCIENCE\_2017\_MGE\_DIV\_UPREG |
| Enrichment Score (ES) | 0.58705735 |
| Normalized Enrichment Score (NES) | 4.5054593 |
| Nominal p-value | 0.0 |
| FDR q-value | 0.0 |
| FWER p-Value | 0.0 |
Table: GSEA Results Summary

  

Fig 1: Enrichment plot: RICHARDS\_NOWAKOWSKI\_SCIENCE\_2017\_MGE\_DIV\_UPREG      
 Profile of the Running ES Score & Positions of GeneSet Members on the Rank Ordered List

  

| PROBE | GENE SYMBOL | GENE\_TITLE | RANK IN GENE LIST | RANK METRIC SCORE | RUNNING ES | CORE ENRICHMENT || 1 | HIST1H1D |  |  | 0 | 2.323 | 0.0068 | Yes |
| 2 | CLSPN |  |  | 1 | 1.984 | 0.0127 | Yes |
| 3 | HIST1H4C |  |  | 2 | 1.902 | 0.0183 | Yes |
| 4 | RRM2 |  |  | 3 | 1.857 | 0.0237 | Yes |
| 5 | ANLN |  |  | 4 | 1.760 | 0.0289 | Yes |
| 6 | HIST1H1B |  |  | 7 | 1.669 | 0.0332 | Yes |
| 7 | TYMS |  |  | 8 | 1.665 | 0.0381 | Yes |
| 8 | MCM10 |  |  | 9 | 1.654 | 0.0430 | Yes |
| 9 | CDC6 |  |  | 11 | 1.631 | 0.0475 | Yes |
| 10 | CDK1 |  |  | 14 | 1.540 | 0.0515 | Yes |
| 11 | DIAPH3 |  |  | 15 | 1.531 | 0.0560 | Yes |
| 12 | ZWINT |  |  | 16 | 1.510 | 0.0605 | Yes |
| 13 | HJURP |  |  | 17 | 1.490 | 0.0648 | Yes |
| 14 | ATAD5 |  |  | 18 | 1.469 | 0.0692 | Yes |
| 15 | FEN1 |  |  | 19 | 1.464 | 0.0735 | Yes |
| 16 | BRCA2 |  |  | 22 | 1.445 | 0.0772 | Yes |
| 17 | UBE2C |  |  | 23 | 1.438 | 0.0814 | Yes |
| 18 | CENPU |  |  | 24 | 1.416 | 0.0855 | Yes |
| 19 | RAD51AP1 |  |  | 25 | 1.384 | 0.0896 | Yes |
| 20 | CENPK |  |  | 26 | 1.382 | 0.0937 | Yes |
| 21 | MYBL2 |  |  | 28 | 1.362 | 0.0974 | Yes |
| 22 | FAM111B |  |  | 29 | 1.361 | 0.1014 | Yes |
| 23 | FBXO5 |  |  | 30 | 1.359 | 0.1054 | Yes |
| 24 | USP1 |  |  | 31 | 1.357 | 0.1094 | Yes |
| 25 | TCF19 |  |  | 32 | 1.331 | 0.1133 | Yes |
| 26 | DTL |  |  | 33 | 1.330 | 0.1172 | Yes |
| 27 | SLC43A3 |  |  | 34 | 1.323 | 0.1211 | Yes |
| 28 | RRM1 |  |  | 37 | 1.310 | 0.1244 | Yes |
| 29 | ORC6 |  |  | 39 | 1.308 | 0.1280 | Yes |
| 30 | GINS2 |  |  | 40 | 1.306 | 0.1318 | Yes |
| 31 | CDCA5 |  |  | 41 | 1.289 | 0.1356 | Yes |
| 32 | TK1 |  |  | 42 | 1.281 | 0.1394 | Yes |
| 33 | AURKB |  |  | 44 | 1.276 | 0.1429 | Yes |
| 34 | MCM7 |  |  | 45 | 1.273 | 0.1466 | Yes |
| 35 | HELLS |  |  | 46 | 1.259 | 0.1503 | Yes |
| 36 | MCM8 |  |  | 48 | 1.253 | 0.1537 | Yes |
| 37 | BRCA1 |  |  | 49 | 1.249 | 0.1574 | Yes |
| 38 | GINS1 |  |  | 52 | 1.239 | 0.1605 | Yes |
| 39 | DNAJC9 |  |  | 56 | 1.217 | 0.1632 | Yes |
| 40 | SKA3 |  |  | 57 | 1.215 | 0.1668 | Yes |
| 41 | PCNA |  |  | 58 | 1.214 | 0.1704 | Yes |
| 42 | CDC45 |  |  | 59 | 1.212 | 0.1739 | Yes |
| 43 | HIST1H1C |  |  | 60 | 1.206 | 0.1775 | Yes |
| 44 | MAD2L1 |  |  | 61 | 1.199 | 0.1810 | Yes |
| 45 | UBE2T |  |  | 62 | 1.192 | 0.1845 | Yes |
| 46 | ATAD2 |  |  | 63 | 1.189 | 0.1880 | Yes |
| 47 | PRIM1 |  |  | 65 | 1.170 | 0.1912 | Yes |
| 48 | MSH2 |  |  | 66 | 1.163 | 0.1946 | Yes |
| 49 | RFC3 |  |  | 67 | 1.157 | 0.1980 | Yes |
| 50 | SPC25 |  |  | 68 | 1.156 | 0.2014 | Yes |
| 51 | MELK |  |  | 69 | 1.145 | 0.2048 | Yes |
| 52 | POLD3 |  |  | 70 | 1.143 | 0.2081 | Yes |
| 53 | WDHD1 |  |  | 71 | 1.133 | 0.2115 | Yes |
| 54 | TOP2A |  |  | 72 | 1.132 | 0.2148 | Yes |
| 55 | KIF2C |  |  | 73 | 1.132 | 0.2181 | Yes |
| 56 | MCM5 |  |  | 74 | 1.128 | 0.2214 | Yes |
| 57 | DSN1 |  |  | 75 | 1.122 | 0.2247 | Yes |
| 58 | CCNE2 |  |  | 76 | 1.121 | 0.2280 | Yes |
| 59 | UHRF1 |  |  | 79 | 1.113 | 0.2308 | Yes |
| 60 | FAM111A |  |  | 80 | 1.112 | 0.2340 | Yes |
| 61 | MCM4 |  |  | 81 | 1.110 | 0.2373 | Yes |
| 62 | RBL1 |  |  | 82 | 1.110 | 0.2406 | Yes |
| 63 | GMNN |  |  | 83 | 1.108 | 0.2438 | Yes |
| 64 | RBBP8 |  |  | 86 | 1.100 | 0.2465 | Yes |
| 65 | HIST1H1E |  |  | 90 | 1.088 | 0.2489 | Yes |
| 66 | SKA1 |  |  | 91 | 1.078 | 0.2520 | Yes |
| 67 | XRCC2 |  |  | 92 | 1.076 | 0.2552 | Yes |
| 68 | VRK1 |  |  | 93 | 1.074 | 0.2584 | Yes |
| 69 | NDC80 |  |  | 94 | 1.074 | 0.2615 | Yes |
| 70 | MASTL |  |  | 96 | 1.063 | 0.2644 | Yes |
| 71 | LRR1 |  |  | 98 | 1.060 | 0.2672 | Yes |
| 72 | TMPO |  |  | 99 | 1.056 | 0.2703 | Yes |
| 73 | NCAPG |  |  | 100 | 1.056 | 0.2734 | Yes |
| 74 | DHFR |  |  | 101 | 1.051 | 0.2765 | Yes |
| 75 | MYBL1 |  |  | 102 | 1.043 | 0.2796 | Yes |
| 76 | MND1 |  |  | 104 | 1.036 | 0.2824 | Yes |
| 77 | MCM3 |  |  | 105 | 1.035 | 0.2854 | Yes |
| 78 | MKI67 |  |  | 107 | 1.031 | 0.2882 | Yes |
| 79 | STIL |  |  | 108 | 1.031 | 0.2912 | Yes |
| 80 | FANCA |  |  | 109 | 1.029 | 0.2942 | Yes |
| 81 | BRIP1 |  |  | 110 | 1.029 | 0.2972 | Yes |
| 82 | TPX2 |  |  | 111 | 1.025 | 0.3003 | Yes |
| 83 | RFC2 |  |  | 112 | 1.022 | 0.3033 | Yes |
| 84 | CDCA8 |  |  | 113 | 1.018 | 0.3063 | Yes |
| 85 | KIF11 |  |  | 115 | 1.014 | 0.3090 | Yes |
| 86 | NCAPH |  |  | 116 | 1.013 | 0.3119 | Yes |
| 87 | NCAPD3 |  |  | 117 | 1.013 | 0.3149 | Yes |
| 88 | DUT |  |  | 118 | 1.009 | 0.3179 | Yes |
| 89 | ASF1B |  |  | 120 | 1.007 | 0.3206 | Yes |
| 90 | SMC2 |  |  | 123 | 1.005 | 0.3230 | Yes |
| 91 | NUF2 |  |  | 125 | 1.004 | 0.3257 | Yes |
| 92 | CHAF1A |  |  | 127 | 0.999 | 0.3283 | Yes |
| 93 | CKS1B |  |  | 128 | 0.999 | 0.3313 | Yes |
| 94 | CENPL |  |  | 132 | 0.989 | 0.3333 | Yes |
| 95 | KIF20B |  |  | 133 | 0.989 | 0.3362 | Yes |
| 96 | MGME1 |  |  | 135 | 0.988 | 0.3389 | Yes |
| 97 | FANCI |  |  | 138 | 0.984 | 0.3412 | Yes |
| 98 | WDR76 |  |  | 142 | 0.975 | 0.3433 | Yes |
| 99 | ESCO2 |  |  | 144 | 0.971 | 0.3458 | Yes |
| 100 | EXO1 |  |  | 145 | 0.969 | 0.3487 | Yes |
| 101 | PLP2 |  |  | 147 | 0.962 | 0.3512 | Yes |
| 102 | CKAP2L |  |  | 148 | 0.959 | 0.3541 | Yes |
| 103 | RANBP1 |  |  | 149 | 0.958 | 0.3569 | Yes |
| 104 | BLM |  |  | 156 | 0.942 | 0.3580 | Yes |
| 105 | NCAPG2 |  |  | 159 | 0.937 | 0.3602 | Yes |
| 106 | KIF23 |  |  | 161 | 0.936 | 0.3627 | Yes |
| 107 | CTPS1 |  |  | 162 | 0.934 | 0.3654 | Yes |
| 108 | SHCBP1 |  |  | 164 | 0.933 | 0.3679 | Yes |
| 109 | RTKN2 |  |  | 166 | 0.931 | 0.3704 | Yes |
| 110 | GTSE1 |  |  | 167 | 0.929 | 0.3731 | Yes |
| 111 | RPA2 |  |  | 169 | 0.929 | 0.3755 | Yes |
| 112 | CDCA2 |  |  | 171 | 0.922 | 0.3780 | Yes |
| 113 | MTHFD1 |  |  | 173 | 0.914 | 0.3804 | Yes |
| 114 | MCM6 |  |  | 176 | 0.906 | 0.3825 | Yes |
| 115 | CENPM |  |  | 178 | 0.901 | 0.3849 | Yes |
| 116 | E2F7 |  |  | 179 | 0.901 | 0.3875 | Yes |
| 117 | C21orf58 |  |  | 201 | 0.873 | 0.3843 | Yes |
| 118 | PLK4 |  |  | 205 | 0.870 | 0.3860 | Yes |
| 119 | CSE1L |  |  | 210 | 0.866 | 0.3875 | Yes |
| 120 | PRIM2 |  |  | 212 | 0.865 | 0.3898 | Yes |
| 121 | H2AFZ |  |  | 213 | 0.864 | 0.3923 | Yes |
| 122 | PBK |  |  | 214 | 0.864 | 0.3948 | Yes |
| 123 | CEP152 |  |  | 217 | 0.860 | 0.3968 | Yes |
| 124 | CDCA3 |  |  | 219 | 0.858 | 0.3991 | Yes |
| 125 | RFC5 |  |  | 222 | 0.851 | 0.4010 | Yes |
| 126 | BUB1B |  |  | 223 | 0.851 | 0.4035 | Yes |
| 127 | CCDC15 |  |  | 230 | 0.845 | 0.4043 | Yes |
| 128 | ASPM |  |  | 231 | 0.842 | 0.4068 | Yes |
| 129 | EZH2 |  |  | 233 | 0.839 | 0.4090 | Yes |
| 130 | HMGA1 |  |  | 238 | 0.834 | 0.4104 | Yes |
| 131 | DBF4 |  |  | 240 | 0.832 | 0.4125 | Yes |
| 132 | RACGAP1 |  |  | 241 | 0.831 | 0.4150 | Yes |
| 133 | PRC1 |  |  | 242 | 0.830 | 0.4174 | Yes |
| 134 | TUBB6 |  |  | 244 | 0.828 | 0.4196 | Yes |
| 135 | C19orf48 |  |  | 248 | 0.826 | 0.4212 | Yes |
| 136 | KNTC1 |  |  | 249 | 0.825 | 0.4236 | Yes |
| 137 | NOP56 |  |  | 250 | 0.824 | 0.4260 | Yes |
| 138 | LMNB1 |  |  | 251 | 0.824 | 0.4285 | Yes |
| 139 | CHEK1 |  |  | 252 | 0.822 | 0.4309 | Yes |
| 140 | NUP155 |  |  | 257 | 0.810 | 0.4321 | Yes |
| 141 | DNA2 |  |  | 263 | 0.797 | 0.4331 | Yes |
| 142 | DNMT1 |  |  | 265 | 0.792 | 0.4352 | Yes |
| 143 | TUBB4B |  |  | 271 | 0.784 | 0.4361 | Yes |
| 144 | HMGB2 |  |  | 276 | 0.780 | 0.4373 | Yes |
| 145 | SMC4 |  |  | 279 | 0.777 | 0.4390 | Yes |
| 146 | DDIAS |  |  | 281 | 0.776 | 0.4410 | Yes |
| 147 | HSPB11 |  |  | 283 | 0.775 | 0.4430 | Yes |
| 148 | CENPE |  |  | 284 | 0.775 | 0.4453 | Yes |
| 149 | TEX30 |  |  | 287 | 0.774 | 0.4470 | Yes |
| 150 | RFWD3 |  |  | 290 | 0.769 | 0.4487 | Yes |
| 151 | BARD1 |  |  | 295 | 0.760 | 0.4499 | Yes |
| 152 | DEK |  |  | 302 | 0.754 | 0.4504 | Yes |
| 153 | POLE |  |  | 305 | 0.750 | 0.4521 | Yes |
| 154 | MMS22L |  |  | 306 | 0.747 | 0.4543 | Yes |
| 155 | CENPO |  |  | 307 | 0.747 | 0.4565 | Yes |
| 156 | KIF4A |  |  | 309 | 0.746 | 0.4584 | Yes |
| 157 | CKS2 |  |  | 312 | 0.745 | 0.4601 | Yes |
| 158 | PRR11 |  |  | 314 | 0.743 | 0.4620 | Yes |
| 159 | ALYREF |  |  | 315 | 0.741 | 0.4641 | Yes |
| 160 | SKP2 |  |  | 317 | 0.740 | 0.4660 | Yes |
| 161 | SLBP |  |  | 318 | 0.740 | 0.4682 | Yes |
| 162 | CENPN |  |  | 320 | 0.737 | 0.4701 | Yes |
| 163 | CCNF |  |  | 321 | 0.737 | 0.4723 | Yes |
| 164 | ANP32B |  |  | 325 | 0.734 | 0.4736 | Yes |
| 165 | IGF2BP1 |  |  | 327 | 0.731 | 0.4755 | Yes |
| 166 | CENPW |  |  | 330 | 0.727 | 0.4771 | Yes |
| 167 | IPO5 |  |  | 334 | 0.724 | 0.4784 | Yes |
| 168 | SPC24 |  |  | 340 | 0.719 | 0.4791 | Yes |
| 169 | LIG1 |  |  | 341 | 0.718 | 0.4812 | Yes |
| 170 | ORC1 |  |  | 342 | 0.717 | 0.4833 | Yes |
| 171 | ARHGAP11A |  |  | 346 | 0.712 | 0.4846 | Yes |
| 172 | AKAP12 |  |  | 349 | 0.711 | 0.4861 | Yes |
| 173 | LMNB2 |  |  | 352 | 0.709 | 0.4877 | Yes |
| 174 | TTK |  |  | 365 | 0.701 | 0.4864 | Yes |
| 175 | POLE2 |  |  | 367 | 0.700 | 0.4882 | Yes |
| 176 | CENPJ |  |  | 376 | 0.695 | 0.4880 | Yes |
| 177 | TMEM106C |  |  | 377 | 0.694 | 0.4901 | Yes |
| 178 | KIF14 |  |  | 378 | 0.693 | 0.4921 | Yes |
| 179 | E2F8 |  |  | 379 | 0.691 | 0.4942 | Yes |
| 180 | SIVA1 |  |  | 380 | 0.690 | 0.4962 | Yes |
| 181 | PARPBP |  |  | 382 | 0.688 | 0.4979 | Yes |
| 182 | RFC4 |  |  | 385 | 0.686 | 0.4994 | Yes |
| 183 | KIF18A |  |  | 392 | 0.683 | 0.4998 | Yes |
| 184 | SUPT16H |  |  | 394 | 0.682 | 0.5015 | Yes |
| 185 | EXOSC9 |  |  | 399 | 0.679 | 0.5024 | Yes |
| 186 | TMEM97 |  |  | 403 | 0.676 | 0.5035 | Yes |
| 187 | KIF15 |  |  | 404 | 0.676 | 0.5055 | Yes |
| 188 | CTNNAL1 |  |  | 405 | 0.676 | 0.5075 | Yes |
| 189 | ZNF367 |  |  | 408 | 0.673 | 0.5089 | Yes |
| 190 | BRIX1 |  |  | 410 | 0.672 | 0.5106 | Yes |
| 191 | CCDC34 |  |  | 411 | 0.672 | 0.5126 | Yes |
| 192 | PA2G4 |  |  | 412 | 0.672 | 0.5146 | Yes |
| 193 | TTF2 |  |  | 413 | 0.671 | 0.5166 | Yes |
| 194 | SAE1 |  |  | 418 | 0.669 | 0.5174 | Yes |
| 195 | FOXM1 |  |  | 423 | 0.667 | 0.5183 | Yes |
| 196 | RAN |  |  | 424 | 0.667 | 0.5203 | Yes |
| 197 | SMC3 |  |  | 425 | 0.667 | 0.5222 | Yes |
| 198 | PMAIP1 |  |  | 426 | 0.666 | 0.5242 | Yes |
| 199 | PHGDH |  |  | 429 | 0.664 | 0.5256 | Yes |
| 200 | RMI1 |  |  | 433 | 0.660 | 0.5267 | Yes |
| 201 | ZWILCH |  |  | 436 | 0.659 | 0.5281 | Yes |
| 202 | BUB3 |  |  | 441 | 0.657 | 0.5289 | Yes |
| 203 | POLA1 |  |  | 443 | 0.654 | 0.5306 | Yes |
| 204 | SLC7A1 |  |  | 448 | 0.651 | 0.5314 | Yes |
| 205 | PARP2 |  |  | 451 | 0.650 | 0.5327 | Yes |
| 206 | RAD54L |  |  | 456 | 0.648 | 0.5335 | Yes |
| 207 | NCAPD2 |  |  | 457 | 0.647 | 0.5354 | Yes |
| 208 | NUDT15 |  |  | 461 | 0.646 | 0.5365 | Yes |
| 209 | RAD18 |  |  | 465 | 0.642 | 0.5376 | Yes |
| 210 | SNRPB |  |  | 467 | 0.642 | 0.5392 | Yes |
| 211 | SSRP1 |  |  | 478 | 0.638 | 0.5383 | Yes |
| 212 | CKLF |  |  | 482 | 0.637 | 0.5393 | Yes |
| 213 | POLH |  |  | 487 | 0.635 | 0.5401 | Yes |
| 214 | FADS1 |  |  | 498 | 0.629 | 0.5392 | Yes |
| 215 | ENO1 |  |  | 501 | 0.629 | 0.5405 | Yes |
| 216 | BIRC5 |  |  | 505 | 0.628 | 0.5415 | Yes |
| 217 | SEPT10 |  |  | 507 | 0.627 | 0.5431 | Yes |
| 218 | IQGAP3 |  |  | 516 | 0.621 | 0.5427 | Yes |
| 219 | GEN1 |  |  | 517 | 0.620 | 0.5445 | Yes |
| 220 | HAUS8 |  |  | 527 | 0.613 | 0.5439 | Yes |
| 221 | CDK2 |  |  | 530 | 0.612 | 0.5451 | Yes |
| 222 | ZGRF1 |  |  | 531 | 0.611 | 0.5469 | Yes |
| 223 | RPA3 |  |  | 532 | 0.611 | 0.5487 | Yes |
| 224 | BTG3 |  |  | 540 | 0.607 | 0.5486 | Yes |
| 225 | DDX39A |  |  | 542 | 0.605 | 0.5501 | Yes |
| 226 | RCC1 |  |  | 549 | 0.603 | 0.5502 | Yes |
| 227 | CENPQ |  |  | 569 | 0.590 | 0.5467 | Yes |
| 228 | MIS18BP1 |  |  | 574 | 0.588 | 0.5473 | Yes |
| 229 | CENPF |  |  | 575 | 0.588 | 0.5490 | Yes |
| 230 | PCNT |  |  | 576 | 0.588 | 0.5508 | Yes |
| 231 | CCNB1 |  |  | 579 | 0.587 | 0.5519 | Yes |
| 232 | IPO7 |  |  | 586 | 0.584 | 0.5520 | Yes |
| 233 | TIMELESS |  |  | 591 | 0.583 | 0.5526 | Yes |
| 234 | TMEM98 |  |  | 594 | 0.580 | 0.5538 | Yes |
| 235 | DDX11 |  |  | 596 | 0.579 | 0.5552 | Yes |
| 236 | CEP135 |  |  | 598 | 0.578 | 0.5566 | Yes |
| 237 | EXOSC8 |  |  | 604 | 0.576 | 0.5569 | Yes |
| 238 | GTPBP4 |  |  | 605 | 0.576 | 0.5586 | Yes |
| 239 | MSH6 |  |  | 611 | 0.574 | 0.5589 | Yes |
| 240 | PPAT |  |  | 616 | 0.570 | 0.5595 | Yes |
| 241 | TUBG1 |  |  | 617 | 0.570 | 0.5612 | Yes |
| 242 | SMC1A |  |  | 618 | 0.570 | 0.5629 | Yes |
| 243 | CKAP2 |  |  | 619 | 0.569 | 0.5645 | Yes |
| 244 | CDC25C |  |  | 626 | 0.568 | 0.5646 | Yes |
| 245 | ADCY3 |  |  | 630 | 0.566 | 0.5654 | Yes |
| 246 | HAT1 |  |  | 632 | 0.565 | 0.5668 | Yes |
| 247 | NEDD1 |  |  | 633 | 0.565 | 0.5684 | Yes |
| 248 | SASS6 |  |  | 637 | 0.562 | 0.5693 | Yes |
| 249 | NCL |  |  | 638 | 0.560 | 0.5709 | Yes |
| 250 | SPAG5 |  |  | 642 | 0.555 | 0.5717 | Yes |
| 251 | HAUS1 |  |  | 649 | 0.552 | 0.5717 | Yes |
| 252 | YEATS4 |  |  | 652 | 0.550 | 0.5728 | Yes |
| 253 | NUSAP1 |  |  | 654 | 0.550 | 0.5741 | Yes |
| 254 | PRPS2 |  |  | 667 | 0.546 | 0.5724 | Yes |
| 255 | PAICS |  |  | 668 | 0.546 | 0.5740 | Yes |
| 256 | H2AFX |  |  | 671 | 0.544 | 0.5750 | Yes |
| 257 | MNS1 |  |  | 672 | 0.543 | 0.5766 | Yes |
| 258 | TUBB |  |  | 678 | 0.538 | 0.5769 | Yes |
| 259 | SLC25A5 |  |  | 680 | 0.537 | 0.5782 | Yes |
| 260 | PRKDC |  |  | 681 | 0.537 | 0.5797 | Yes |
| 261 | WEE1 |  |  | 685 | 0.536 | 0.5805 | Yes |
| 262 | CIT |  |  | 690 | 0.535 | 0.5810 | Yes |
| 263 | ARF6 |  |  | 697 | 0.533 | 0.5809 | Yes |
| 264 | HADH |  |  | 706 | 0.530 | 0.5802 | Yes |
| 265 | RIF1 |  |  | 713 | 0.529 | 0.5801 | Yes |
| 266 | CORO1C |  |  | 718 | 0.526 | 0.5806 | Yes |
| 267 | GPSM2 |  |  | 719 | 0.526 | 0.5821 | Yes |
| 268 | BAZ1B |  |  | 721 | 0.525 | 0.5834 | Yes |
| 269 | HAUS6 |  |  | 724 | 0.525 | 0.5844 | Yes |
| 270 | CCT6A |  |  | 725 | 0.525 | 0.5859 | Yes |
| 271 | C4orf46 |  |  | 733 | 0.521 | 0.5855 | Yes |
| 272 | MCM2 |  |  | 734 | 0.521 | 0.5871 | Yes |
| 273 | NASP |  |  | 741 | 0.518 | 0.5869 | No |
| 274 | LINC01224 |  |  | 760 | 0.513 | 0.5835 | No |
| 275 | SNX5 |  |  | 762 | 0.512 | 0.5847 | No |
| 276 | POLQ |  |  | 771 | 0.508 | 0.5840 | No |
| 277 | NUP62 |  |  | 777 | 0.505 | 0.5841 | No |
| 278 | CMC2 |  |  | 785 | 0.503 | 0.5837 | No |
| 279 | CCDC150 |  |  | 804 | 0.496 | 0.5802 | No |
| 280 | AHI1 |  |  | 814 | 0.494 | 0.5791 | No |
| 281 | SRSF10 |  |  | 831 | 0.489 | 0.5762 | No |
| 282 | ACYP1 |  |  | 834 | 0.489 | 0.5770 | No |
| 283 | UBR7 |  |  | 857 | 0.483 | 0.5724 | No |
| 284 | RND3 |  |  | 860 | 0.482 | 0.5733 | No |
| 285 | TUBA1B |  |  | 862 | 0.481 | 0.5744 | No |
| 286 | ARL6IP6 |  |  | 880 | 0.476 | 0.5711 | No |
| 287 | PNN |  |  | 882 | 0.476 | 0.5722 | No |
| 288 | CASP2 |  |  | 884 | 0.474 | 0.5734 | No |
| 289 | DERA |  |  | 886 | 0.474 | 0.5745 | No |
| 290 | NUP50 |  |  | 888 | 0.473 | 0.5756 | No |
| 291 | CISD2 |  |  | 891 | 0.473 | 0.5764 | No |
| 292 | SUZ12 |  |  | 894 | 0.472 | 0.5773 | No |
| 293 | CEP57L1 |  |  | 901 | 0.471 | 0.5770 | No |
| 294 | DEPDC1B |  |  | 906 | 0.470 | 0.5773 | No |
| 295 | ZNF90 |  |  | 921 | 0.466 | 0.5748 | No |
| 296 | ITGB3BP |  |  | 935 | 0.462 | 0.5726 | No |
| 297 | HIRIP3 |  |  | 936 | 0.462 | 0.5739 | No |
| 298 | SRSF2 |  |  | 941 | 0.461 | 0.5742 | No |
| 299 | LIN9 |  |  | 949 | 0.459 | 0.5736 | No |
| 300 | BAZ1A |  |  | 960 | 0.455 | 0.5722 | No |
| 301 | RAD51C |  |  | 967 | 0.453 | 0.5719 | No |
| 302 | CDC7 |  |  | 971 | 0.451 | 0.5724 | No |
| 303 | KIF22 |  |  | 975 | 0.450 | 0.5729 | No |
| 304 | FANCD2 |  |  | 976 | 0.450 | 0.5742 | No |
| 305 | MIS12 |  |  | 1018 | 0.439 | 0.5642 | No |
| 306 | SEC11A |  |  | 1019 | 0.438 | 0.5655 | No |
| 307 | CASP8AP2 |  |  | 1025 | 0.436 | 0.5654 | No |
| 308 | TOMM5 |  |  | 1049 | 0.428 | 0.5603 | No |
| 309 | FANCL |  |  | 1052 | 0.427 | 0.5610 | No |
| 310 | DLEU2 |  |  | 1059 | 0.426 | 0.5606 | No |
| 311 | SRSF7 |  |  | 1071 | 0.424 | 0.5588 | No |
| 312 | NUDT21 |  |  | 1076 | 0.422 | 0.5590 | No |
| 313 | JADE1 |  |  | 1089 | 0.419 | 0.5569 | No |
| 314 | KCTD9 |  |  | 1104 | 0.416 | 0.5542 | No |
| 315 | CTDSPL2 |  |  | 1109 | 0.415 | 0.5544 | No |
| 316 | TPI1 |  |  | 1111 | 0.415 | 0.5553 | No |
| 317 | CYCS |  |  | 1113 | 0.415 | 0.5563 | No |
| 318 | RCC2 |  |  | 1124 | 0.413 | 0.5547 | No |
| 319 | HNRNPF |  |  | 1128 | 0.412 | 0.5551 | No |
| 320 | CBX5 |  |  | 1132 | 0.412 | 0.5555 | No |
| 321 | POLR3K |  |  | 1140 | 0.410 | 0.5548 | No |
| 322 | CNTLN |  |  | 1142 | 0.409 | 0.5557 | No |
| 323 | HSPA14 |  |  | 1148 | 0.408 | 0.5555 | No |
| 324 | HNRNPD |  |  | 1177 | 0.401 | 0.5490 | No |
| 325 | SNRPE |  |  | 1185 | 0.399 | 0.5482 | No |
| 326 | SACS |  |  | 1195 | 0.397 | 0.5469 | No |
| 327 | NUP43 |  |  | 1212 | 0.394 | 0.5437 | No |
| 328 | THOC1 |  |  | 1224 | 0.392 | 0.5418 | No |
| 329 | PTMA |  |  | 1233 | 0.390 | 0.5407 | No |
| 330 | VPS29 |  |  | 1234 | 0.390 | 0.5419 | No |
| 331 | STRIP2 |  |  | 1236 | 0.389 | 0.5427 | No |
| 332 | PSMC3 |  |  | 1241 | 0.389 | 0.5428 | No |
| 333 | STMN1 |  |  | 1249 | 0.387 | 0.5420 | No |
| 334 | ZNF93 |  |  | 1265 | 0.384 | 0.5390 | No |
| 335 | ERLIN1 |  |  | 1274 | 0.382 | 0.5379 | No |
| 336 | RNF138 |  |  | 1278 | 0.381 | 0.5382 | No |
| 337 | TTL |  |  | 1289 | 0.380 | 0.5366 | No |
| 338 | SNRPD3 |  |  | 1303 | 0.377 | 0.5341 | No |
| 339 | HMGXB4 |  |  | 1322 | 0.375 | 0.5302 | No |
| 340 | STK17B |  |  | 1324 | 0.374 | 0.5311 | No |
| 341 | RAD54B |  |  | 1327 | 0.373 | 0.5316 | No |
| 342 | ANP32E |  |  | 1334 | 0.371 | 0.5310 | No |
| 343 | RNASEH2A |  |  | 1341 | 0.369 | 0.5305 | No |
| 344 | FDPS |  |  | 1348 | 0.367 | 0.5299 | No |
| 345 | ZNF829 |  |  | 1350 | 0.367 | 0.5307 | No |
| 346 | TOPBP1 |  |  | 1355 | 0.366 | 0.5307 | No |
| 347 | SHMT2 |  |  | 1361 | 0.366 | 0.5304 | No |
| 348 | LDHA |  |  | 1374 | 0.361 | 0.5281 | No |
| 349 | ACP1 |  |  | 1396 | 0.358 | 0.5234 | No |
| 350 | SLC20A1 |  |  | 1400 | 0.357 | 0.5236 | No |
| 351 | ANKRD36C |  |  | 1404 | 0.357 | 0.5238 | No |
| 352 | SENP1 |  |  | 1408 | 0.356 | 0.5241 | No |
| 353 | SPATA5 |  |  | 1424 | 0.352 | 0.5210 | No |
| 354 | PARP1 |  |  | 1431 | 0.351 | 0.5203 | No |
| 355 | IMMT |  |  | 1436 | 0.350 | 0.5203 | No |
| 356 | HAUS5 |  |  | 1463 | 0.345 | 0.5141 | No |
| 357 | THOC3 |  |  | 1485 | 0.342 | 0.5093 | No |
| 358 | NRM |  |  | 1550 | 0.331 | 0.4927 | No |
| 359 | CLIC1 |  |  | 1552 | 0.331 | 0.4934 | No |
| 360 | ACTB |  |  | 1565 | 0.330 | 0.4910 | No |
| 361 | TIMM10 |  |  | 1567 | 0.329 | 0.4917 | No |
| 362 | WWOX |  |  | 1568 | 0.329 | 0.4927 | No |
| 363 | TMEM237 |  |  | 1576 | 0.328 | 0.4917 | No |
| 364 | TPM4 |  |  | 1578 | 0.328 | 0.4924 | No |
| 365 | NEDD4 |  |  | 1622 | 0.320 | 0.4815 | No |
| 366 | CALM2 |  |  | 1625 | 0.320 | 0.4819 | No |
| 367 | SF3B3 |  |  | 1651 | 0.316 | 0.4760 | No |
| 368 | LINC00342 |  |  | 1685 | 0.312 | 0.4678 | No |
| 369 | RNASEH2C |  |  | 1697 | 0.311 | 0.4657 | No |
| 370 | TMX1 |  |  | 1703 | 0.310 | 0.4652 | No |
| 371 | IPO9 |  |  | 1705 | 0.310 | 0.4658 | No |
| 372 | HMGB1 |  |  | 1720 | 0.308 | 0.4629 | No |
| 373 | TARS |  |  | 1730 | 0.307 | 0.4613 | No |
| 374 | ENOSF1 |  |  | 1823 | 0.296 | 0.4368 | No |
| 375 | CNTRL |  |  | 1830 | 0.294 | 0.4360 | No |
| 376 | ILF2 |  |  | 1833 | 0.294 | 0.4363 | No |
| 377 | SRSF3 |  |  | 1834 | 0.294 | 0.4372 | No |
| 378 | DIS3L |  |  | 1865 | 0.290 | 0.4298 | No |
| 379 | PRTFDC1 |  |  | 1866 | 0.290 | 0.4306 | No |
| 380 | GLO1 |  |  | 1867 | 0.290 | 0.4315 | No |
| 381 | STK39 |  |  | 1871 | 0.289 | 0.4315 | No |
| 382 | MTAP |  |  | 1937 | 0.281 | 0.4144 | No |
| 383 | PSMB2 |  |  | 1950 | 0.279 | 0.4120 | No |
| 384 | SRRM1 |  |  | 1958 | 0.278 | 0.4108 | No |
| 385 | E2F3 |  |  | 1961 | 0.277 | 0.4111 | No |
| 386 | PPIE |  |  | 1965 | 0.277 | 0.4111 | No |
| 387 | TRIM26 |  |  | 2005 | 0.272 | 0.4011 | No |
| 388 | KITLG |  |  | 2019 | 0.270 | 0.3984 | No |
| 389 | ERI2 |  |  | 2034 | 0.269 | 0.3953 | No |
| 390 | EEF1B2 |  |  | 2041 | 0.268 | 0.3944 | No |
| 391 | SKA2 |  |  | 2061 | 0.266 | 0.3900 | No |
| 392 | ADK |  |  | 2069 | 0.265 | 0.3888 | No |
| 393 | PPIA |  |  | 2072 | 0.265 | 0.3891 | No |
| 394 | MPHOSPH9 |  |  | 2082 | 0.264 | 0.3873 | No |
| 395 | CEP192 |  |  | 2109 | 0.262 | 0.3810 | No |
| 396 | RAP1B |  |  | 2118 | 0.261 | 0.3795 | No |
| 397 | CKAP5 |  |  | 2135 | 0.258 | 0.3759 | No |
| 398 | XRCC6 |  |  | 2180 | 0.254 | 0.3645 | No |
| 399 | OXCT1 |  |  | 2185 | 0.253 | 0.3641 | No |
| 400 | SEPHS1 |  |  | 2188 | 0.253 | 0.3643 | No |
| 401 | PCBP1 |  |  | 2192 | 0.253 | 0.3642 | No |
| 402 | PKM |  |  | 2199 | 0.252 | 0.3633 | No |
| 403 | IQCB1 |  |  | 2215 | 0.250 | 0.3599 | No |
| 404 | PCDH19 |  |  | 2279 | -0.257 | 0.3433 | No |
| 405 | RAB13 |  |  | 2381 | -0.268 | 0.3163 | No |
| 406 | PSME1 |  |  | 2413 | -0.272 | 0.3085 | No |
| 407 | SRP9 |  |  | 2441 | -0.276 | 0.3019 | No |
| 408 | ILDR2 |  |  | 2478 | -0.280 | 0.2928 | No |
| 409 | SEPT2 |  |  | 2482 | -0.281 | 0.2928 | No |
| 410 | RPL3 |  |  | 2613 | -0.303 | 0.2579 | No |
| 411 | SRGAP2B |  |  | 2638 | -0.307 | 0.2522 | No |
| 412 | ANKRD50 |  |  | 2653 | -0.309 | 0.2492 | No |
| 413 | EP300 |  |  | 2670 | -0.312 | 0.2457 | No |
| 414 | APRT |  |  | 2680 | -0.313 | 0.2442 | No |
| 415 | C12orf57 |  |  | 2698 | -0.316 | 0.2404 | No |
| 416 | BROX |  |  | 2730 | -0.322 | 0.2328 | No |
| 417 | ZNF43 |  |  | 2872 | -0.348 | 0.1950 | No |
| 418 | FIGN |  |  | 2879 | -0.351 | 0.1944 | No |
| 419 | PCDH17 |  |  | 2924 | -0.360 | 0.1833 | No |
| 420 | APBB2 |  |  | 3023 | -0.378 | 0.1574 | No |
| 421 | C6orf62 |  |  | 3042 | -0.384 | 0.1536 | No |
| 422 | ABAT |  |  | 3075 | -0.391 | 0.1459 | No |
| 423 | AGL |  |  | 3134 | -0.410 | 0.1311 | No |
| 424 | TMSB4X |  |  | 3141 | -0.412 | 0.1307 | No |
| 425 | HMGN5 |  |  | 3173 | -0.421 | 0.1234 | No |
| 426 | PDZRN3 |  |  | 3276 | -0.449 | 0.0966 | No |
| 427 | BEST3 |  |  | 3302 | -0.458 | 0.0911 | No |
| 428 | DAZAP2 |  |  | 3319 | -0.463 | 0.0880 | No |
| 429 | HSPA5 |  |  | 3335 | -0.468 | 0.0853 | No |
| 430 | GLCCI1 |  |  | 3444 | -0.517 | 0.0570 | No |
| 431 | CDK6 |  |  | 3466 | -0.526 | 0.0528 | No |
| 432 | H3F3A |  |  | 3471 | -0.528 | 0.0532 | No |
| 433 | HUNK |  |  | 3569 | -0.582 | 0.0282 | No |
| 434 | DBI |  |  | 3597 | -0.601 | 0.0225 | No |
| 435 | FBXW7 |  |  | 3629 | -0.626 | 0.0158 | No |
| 436 | TFDP2 |  |  | 3637 | -0.634 | 0.0158 | No |
| 437 | CDCA7L |  |  | 3642 | -0.637 | 0.0165 | No |
| 438 | SH3BGRL |  |  | 3663 | -0.654 | 0.0129 | No |
| 439 | LITAF |  |  | 3670 | -0.660 | 0.0132 | No |
| 440 | DCHS1 |  |  | 3674 | -0.662 | 0.0144 | No |
| 441 | SERINC5 |  |  | 3676 | -0.664 | 0.0160 | No |
| 442 | MDK |  |  | 3706 | -0.691 | 0.0101 | No |
| 443 | PET100 |  |  | 3727 | -0.706 | 0.0066 | No |
| 444 | TMA7 |  |  | 3731 | -0.710 | 0.0079 | No |
| 445 | DLX6 |  |  | 3735 | -0.711 | 0.0092 | No |
| 446 | NRXN3 |  |  | 3738 | -0.714 | 0.0107 | No |
| 447 | ZNF704 |  |  | 3750 | -0.729 | 0.0098 | No |
| 448 | RIC3 |  |  | 3767 | -0.757 | 0.0076 | No |
| 449 | COX6C |  |  | 3792 | -0.782 | 0.0033 | No |
| 450 | SPECC1 |  |  | 3801 | -0.790 | 0.0034 | No |
| 451 | MSI2 |  |  | 3816 | -0.802 | 0.0019 | No |
| 452 | MAGI1 |  |  | 3827 | -0.815 | 0.0016 | No |
| 453 | RHOBTB3 |  |  | 3838 | -0.829 | 0.0013 | No |
| 454 | PFN2 |  |  | 3841 | -0.832 | 0.0032 | No |
| 455 | SALL1 |  |  | 3863 | -0.855 | -0.0001 | No |
| 456 | ZEB2 |  |  | 3876 | -0.894 | -0.0008 | No |
| 457 | CHD7 |  |  | 3880 | -0.904 | 0.0010 | No |
| 458 | PROM1 |  |  | 3892 | -0.929 | 0.0008 | No |
| 459 | FBLN1 |  |  | 3909 | -0.965 | -0.0008 | No |
| 460 | SEMA5A |  |  | 3921 | -1.006 | -0.0009 | No |
| 461 | BCHE |  |  | 3926 | -1.012 | 0.0010 | No |
| 462 | RAB3IP |  |  | 3940 | -1.037 | 0.0004 | No |
| 463 | SOX2 |  |  | 3953 | -1.069 | 0.0003 | No |
| 464 | HES6 |  |  | 3961 | -1.092 | 0.0016 | No |
| 465 | PRDX2 |  |  | 4021 | -1.396 | -0.0106 | No |
| 466 | DLX1 |  |  | 4041 | -1.558 | -0.0112 | No |
| 467 | DLX2 |  |  | 4055 | -1.704 | -0.0098 | No |
| 468 | ASCL1 |  |  | 4061 | -1.762 | -0.0060 | No |
| 469 | CKB |  |  | 4075 | -2.149 | -0.0033 | No |
| 470 | LMO1 |  |  | 4093 | -3.174 | 0.0014 | No |
Table: GSEA details [plain text format]

  

Fig 2: RICHARDS\_NOWAKOWSKI\_SCIENCE\_2017\_MGE\_DIV\_UPREG: Random ES distribution      
 Gene set null distribution of ES for **RICHARDS\_NOWAKOWSKI\_SCIENCE\_2017\_MGE\_DIV\_UPREG**
